# Nuclease Activity of the Junín Virus Nucleoprotein C-Terminal Domain

**DOI:** 10.3390/v15091818

**Published:** 2023-08-26

**Authors:** Alicia Armella Sierra, María Eugenia Loureiro, Sebastián Esperante, Silvia Susana Borkosky, Giovanna L. Gallo, Gonzalo de Prat Gay, Nora Lopez

**Affiliations:** 1 Centro de Virología Humana y Animal (CEVHAN), Consejo Nacional de Investigaciones Científicas y Técnicas (CONICET)-Universidad Abierta Interamericana, Buenos Aires C1287, Argentina; alsierrabea@gmail.com (A.A.S.); eugenialoureiro@conicet.gov.ar (M.E.L.); giovannalgallo@gmail.com (G.L.G.); 2 Fundación Instituto Leloir, Instituto de Investigaciones Bioquímicas de Buenos Aires (IIBBA) CONICET, Buenos Aires C1405, Argentina; sesperante@iib.unsam.edu.ar (S.E.); sborkosky@leloir.org.ar (S.S.B.); gpg@leloir.org.ar (G.d.P.G.)

**Keywords:** Junín virus, nucleoprotein, exoribonuclease activity, biophysical characterization

## Abstract

The mammarenavirus Junín (JUNV) is the causative agent of Argentine hemorrhagic fever, a severe disease of public health concern. The most abundant viral protein is the nucleoprotein (NP), a multifunctional, two-domain protein with the primary role as structural component of the viral nucleocapsids, used as template for viral polymerase RNA synthesis activities. Here, we report that the C-terminal domain (CTD) of the attenuated Candid#1 strain of the JUNV NP can be purified as a stable soluble form with a secondary structure in line with known NP structures from other mammarenaviruses. We show that the JUNV NP CTD interacts with the viral matrix protein Z in vitro, and that the full-length NP and Z interact with each other in cellulo, suggesting that the NP CTD is responsible for this interaction. This domain comprises an arrangement of four acidic residues and a histidine residue conserved in the active site of exoribonucleases belonging to the DEDDh family. We show that the JUNV NP CTD displays metal-ion-dependent nuclease activity against DNA and single- and double-stranded RNA, and that this activity is impaired by the mutation of a catalytic residue within the DEDDh motif. These results further support this activity, not previously observed in the JUNV NP, which could impact the mechanism of the cellular immune response modulation of this important pathogen.

## 1. Introduction

The *Arenaviridae* family includes pathogens that cause severe hemorrhagic fever in humans, with high morbidity and mortality rates. Arenaviruses are currently classified into four genera, among which the *Mammarenavirus* genus comprises mammal-infecting viruses. Mammarenaviruses are further divided into Old World (OW) and New World (NW) groups according to geographical distribution, antigenic properties, and phylogenetic relationships [1]. OW mammarenaviruses include the prototypic lymphocytic choriomeningitis virus (LCMV), of worldwide distribution, as well as viruses causing hemorrhagic disease, such as Lassa virus (LASV), which is endemic to West Africa. The NW group encompasses highly pathogenic members endemic to South America, such as Junín virus (JUNV), the causative agent of Argentine hemorrhagic fever (AHF), as well as nonpathogenic viruses including Tacaribe virus (TCRV), a prototypic model of the NW group.

As all mammarenaviruses, JUNV is enveloped and it replicates in the cytoplasm of the infected cells. It contains a negative-sense RNA genome consisting of two single-stranded segments named S (for Small, 3.4 kb) and L (for Large, 7.2 kb), each of which encodes two proteins. The S segment encodes the nucleoprotein (NP) and the glycoprotein precursor (GPC), which is cleaved by cellular proteases to conform the mature envelope glycoprotein complex (GP). GP is responsible for cellular receptor recognition and entry into the target cell [2,3]. The L segment encodes the viral RNA-dependent RNA polymerase (L) and the matrix protein (Z) that directs viral morphogenesis [4].

The NP displays multifunctional roles throughout the mammarenavirus life cycle. It tightly associates with genomic and antigenomic RNAs to form ribonucleoprotein complexes (RNPs) [5], which interact with the L polymerase. The RNP–L assembly constitutes the functional unit that directs the viral replication and transcription processes [6,7,8]. Additionally, it has been demonstrated for TCRV, LASV, and the OW Mopeia virus (MOPV) that the NP interacts with the Z protein, this interaction being essential for the recruitment of RNPs into infectious particles [9,10,11]. In addition, the NP has also been identified as a type I-interferon (IFN-I) antagonist acting at different steps of the IFN-I pathway [12,13,14,15].

To date, no crystal structure of a full-length JUNV NP is available. However, the crystal structure of full-length NP from OW LASV revealed two globular domains: the amino-terminal domain (NTD) and the carboxyl-terminal domain (CTD), which are connected by a flexible linker [16]. The NTD binds single-stranded (ss) RNA and is required for NP homo-oligomerization [17,18,19]. Moreover, we have previously demonstrated that the self-association capacity of the TCRV NP and its interaction with RNA is critical for RNP assembly and the synthesis of viral RNA by the L polymerase [20].

Further crystallographic studies on the CTD from LASV, JUNV, TCRV, LCMV and MOPV NPs [21,22,23,24,25] revealed structural similarity with the DEDDh family of exonucleases (ExoN) found in bacterial and mammalian cells. Proteins of this family are characterized by four invariant acidic residues (Asp-Glu-Asp-Asp) plus an additional conserved His residue that are arranged in a DEDDh motif, which conforms the active site and is essential for ExoN catalytic activity [26]. Indeed, in vitro studies demonstrated that, like other exoribonucleases belonging to the DEDDh family, the CTD from several arenavirus NPs displays 3′-5’ exoribonuclease activity depending on two metal ions, such as Mn^2+^, Co^2+^ and/or Mg^2+^ [16,21,22,24,25,27]. The integrity of the DEDDh motif has been associated with the ability of several arenavirus NPs to suppress IFN-I production [13,14,15,16,21,22,28,29]. A zinc-binding site located near the exonuclease active site, which is thought to stabilize the overall structure of the domain, has been described for the NP CTD from LASV, LCMV, and TCRV [16,19,21,22,24].

The JUNV NP binds zinc ions in vitro [30], and displays structural elements conserved in the NP from other arenaviruses, including the zinc-finger and the DEDDh motifs [23]. However, ExoN activity for the CTD of the JUNV NP was not previously demonstrated. In this work, we present a physicochemical and functional characterization of purified CTD from the JUNV NP. Our results complete the landscape of the JUNV NP functions, showing that the CTD is involved in the interaction of NP with the matrix Z protein. Also, our results clearly demonstrate that the JUNV NP CTD displays ribonuclease activity that is strictly dependent on metal ions and, at least, on one catalytic residue of the DEDDh motif, conserved in all members of the DEDDh family of exoribonucleases. Viral-encoded ribonuclease activities are at the center of replication and host immune response regulation and may provide an antiviral target to control pathogenic mammarenavirus infections.

## 2. Materials and Methods

### 2.1. Cells

CV1 cells were grown in a 5% CO_2_ atmosphere at 37 °C by using Dulbecco’s modified Eagle’s medium (Invitrogen, Waltham, MA, USA), supplemented with 10% fetal calf serum and penicillin (100 U/mL)–streptomycin (100 µg/mL) (Invitrogen, Waltham, MA USA).

### 2.2. Plasmids

Plasmid pMAL-NP_CTD JUNV expresses the NP CTD from the attenuated Candid#1 strain of JUNV (hereafter called NP_CTD) fused at its amino terminus to Maltose Binding Protein (MBP). To construct the pMAL-NP_CTD JUNV, the sequence encoding the CTD of the JUNV NP was amplified using reverse transcription (RT) followed by a polymerase chain reaction (PCR) using total RNA extracted from JUNV Candid#1 strain-infected Vero cells (kindly provided by Ricardo Gómez, Instituto de Biotecnología y Biología Molecular, Argentina), and oligonucleotides designed on the basis of the reported sequence (GenBank accession number AY746353). The forward oligonucleotide (CTD-NJunv Fw, Table 1) included an ATG codon and was 5′ phosphorylated. The reverse oligonucleotide (NJunv Rv, Table 1) comprised an EcoRI site. The 707 bp-long PCR product was purified using agarose gel electrophoresis and then digested with the EcoRI enzyme. The vector, a modified version of pMALp2 containing a thrombin protease cleavage site [31], was digested with BamHI, followed by treatment with Klenow enzyme to fill in the ends, and then digested with EcoRI. The purified PCR fragment and vector were ligated using T4 DNA ligase.

Plasmid pMAL-Z JUNV expresses JUNV Z fused at its amino terminus to the MBP. To obtain it, pTM1-Z JUNV encoding Z JUNV [9] was digested with NcoI, followed by treatment with Klenow, and subsequently digested with SmaI. The fragment of interest (290 bp) was inserted into the modified vector pMALp2 [31], previously digested with EcoRI and BamHI and subsequently treated with Klenow to fill in the ends.

Plasmid pTM1-Z-HA JUNV, expressing JUNV Z fused at its C-terminus with an influenza virus hemagglutinin (HA) epitope (YPYDVPDYA), was previously generated [9].

Plasmid pTM1-Flag-NP JUNV encodes the full-length JUNV NP tagged at its N-terminus with the Flag epitope (DYKDDDDK). For its construction, the JUNV Candid#1 NP coding sequence was amplified by RT-PCR, using a 5′-phosphorylated forward oligonucleotide (Flag-NJunv Fw, Table 1), which included an ATG codon followed by the sequence corresponding to the Flag epitope, and primer NJunv Rv (Table 1). The PCR product was purified using agarose gel electrophoresis and then digested with EcoRI. The pTM1 vector was digested with NcoI, followed by treatment with Klenow to fill in the ends, and then digested with EcoRI. The ligation of the vector and the purified PCR product was carried out using T4 DNA ligase.

A point change was introduced into the pMAL-NP_CTD using a QuickChange PCR mutagenesis kit (Agilent, Santa Clara, CA, USA) with primers containing the mutated sequence, namely NjunvD529A Fw and NjunvD529A Rv (Table 1). Three clones were selected for nucleotide sequencing; the selected mutant plasmid was designated as pMAL NP_CTD D529A.

### 2.3. DNA Purification and Sequencing

The bacterial strain *Escherichia coli* SURE (Stop Unwanted Rearrangement Events, provided by Agilent, Santa Clara, CA, USA) was used for the cloning and amplification of plasmid DNA.

The plasmids were purified using a QIAGEN Plasmid Midi Kit (Qiagen, Germantown, MD, USA) or the Wizard^®^ Plus SV Minipreps DNA Purification System kit (Promega, Madison, WI, USA). The purification of DNA fragments from agarose gels was carried out using the Wizard^®^ SV Gel and PCR Clean-Up System (Promega, Madison, WI, USA).

All the constructions were analyzed using automated dideoxynucleotide DNA sequencing (Macrogen, Seoul, Republic of Korea). Sequence analysis was performed using the Sequence Manipulation Suite tool (https://www.bioinformatics.org/sms/, accessed on 1 June 2022); alignments were carried out using the ClustalX program (http://www.clustal.org/, accessed on 1 June 2022). 

### 2.4. Expression and Purification of Proteins

A fresh colony of *Escherichia coli* Rosetta (DE3) transformed with either pMAL-NP_CTD JUNV or pMAL-Z JUNV was inoculated into 10 mL of Luria broth (LB) medium containing 100 μg/mL ampicillin, 34 μg/mL chloramphenicol and 12.5 μg/mL tetracycline. After incubation for 18 h at 37 °C, the culture was diluted at 1:100 in LB medium and incubated for 3 h at 37 °C, followed by 1 h at 20 °C with continuous shaking at 210 rpm. Protein expression was induced by adding 0.1 mM Isopropyl-β-D-thiogalactoside (IPTG) at OD600 = 0.6 and incubation was continued for 18 h at 20 °C. Cells were harvested via centrifugation at 3800× *g* for 10 min at 4 °C. The bacterial pellet was resuspended in a lysis buffer (100 mM Tris-HCl pH 8; 300 mM NaCl; 1 mM phenylmethylsulfonyl fluoride), lysed via sonication, and centrifuged. Subsequently, solid ammonium sulfate was added to the soluble fraction up to 60% saturation. The precipitated protein was collected via centrifugation at 27,000× *g* for 30 min at 4 °C. The resulting pellet was resuspended in the lysis buffer containing 10 mM Dithiothreitol (DTT), followed by dialysis against a buffer containing 20 mM Tris-HCl (pH 8.0), 400 mM NaCl, and 10 μM ZnCl_2_, for 18 h at 4 °C. Finally, the soluble fraction was subjected to affinity chromatography using amylose resin, according to the manufacturer’s instructions (New England Biolabs, Ipswich, MA, USA).

Purified MBP-NP_CTD (obtained as indicated above) was treated with bovine thrombin (Sigma-Aldrich, Burlington, MA, USA) overnight at 4 °C, to separate the NP_CTD from the MBP tag. The NP_CTD was purified by Anion Exchange chromatography using a HiTrap Q HP column (GE Healthcare), in an ÄKTA^TM^ Purifier. Binding was achieved in a start buffer (20 mM sodium phosphate pH 5.5; 25 mM NaCl) and elution was carried out applying a continuous 0–100% gradient of elution buffer (20 mM sodium phosphate pH 5.5; 1 M NaCl), at a flow rate of 1 mL/min. Fractions corresponding to the absorbance peak at 280 nm were collected. The soluble proteins were concentrated in phosphate buffered saline (PBS) using Amicon^®^ Ultra-15 Centrifugal Filter Units (Sigma-Aldrich, Burlington, MA, USA) and protein quantification was determined using the Bradford method [32]. A ratio of 260:280 nm of ~0.55 for the purified protein was indicative of minimum nucleic acid contamination. 

### 2.5. Determination of Molecular Weight (MW)

Size-exclusion chromatography was carried out on a Superdex 200 HR 10/300 column (GE Healthcare, Chicago, IL, USA). The column was calibrated with the following standard globular proteins: ferritin (440 kDa), catalase (232 kDa), human albumin (67 kDa), ovalbumin (43 kDa), and chymotrypsinogen A (25 kDa) (Sigma-Aldrich, Burlington, MA, USA). The void volume (V0) and total volume (V0 + Vi) were determined with Blue Dextran and acetone, respectively. 

The molecular weight of NP_CTD in solution (26,079 Da), determined by using mass spectrometry (LANAIS PROEM, IQUIFIB-CONICET, CABA, Argentina), agreed with the theoretical molecular weight (26,066.07 Da) calculated through the pI/MW server (https://www.expasy.org/resources/compute-pi-mw, accessed on 1 June 2022).

### 2.6. Circular Dichroism (CD)

Far-UV CD measurements were conducted on a Jasco J-810 spectropolarimeter. The temperature was kept at 20 °C using a Peltier temperature-controlled cell. Spectra were recorded between 195 and 260 nm at standard sensitivity, at a rate of 100 nm/min, a response time of 4 s, a data pitch of 0.1 nm, and a bandwidth of 8 nm. All spectra were an average of at least 6 scans. Protein spectra at 10 μM were taken on a 0.1 cm path-length cell. The ellipticity at 260 nm was subtracted from the other ellipticities as a baseline value. Raw data were converted to molar ellipticity using the following equation

[θ]MRW = deg/([c] × #bonds × L × 10,000)

where deg is the raw signal in millidegs, [c] is the protein concentration in molar units, #bonds is the number of peptide bonds (number of amino acids − 1), and L is the path length in cm.

### 2.7. Protein Analysis

Protein extracts were adjusted to a 1× final concentration of Laemmli buffer (100 mM Tris-HCl pH 6.8, 4% SDS, 20% glycerol, 0.2% bromophenol blue and 4% β-mercaptoethanol). Samples were then treated for 5 min at 95 °C followed by centrifugation for 2 min at 12,000× *g*. Proteins were analyzed using sodium dodecyl sulfate–polyacrylamide gel electrophoresis (SDS-PAGE); commercial molecular weight markers were resolved in parallel. When indicated, proteins were visualized using Coomassie blue staining.

For the Western blotting analysis, proteins resolved by SDS-PAGE were transferred to a Hybond ECL Nitrocellulose membrane (GE Healthcare), which was then stained with Ponceau red (1%), to control for transfer efficiency. Once washed, the membrane was blocked for 1 h at room temperature in PBS containing 0.1% Tween 20 (PBS-T), and 5% skim milk. Subsequently, blots were incubated with a dilution of the primary antibody in PBS-T containing 3% skim milk, for 16 h at 4 °C, with orbital shaking. After three washes with PBS-T for 10 min each, blots were incubated with an anti-rabbit or anti-mouse peroxidase-conjugated secondary antibody (Jackson ImmunoResearch, West Grove, PA, USA) (1:5000) for 1 h at room temperature. After washing the membrane exhaustively with PBS-T, the proteins were detected via chemiluminescence using a Western Detection Reagent (Pierce, Thermo Fisher, Waltham, MA, USA) on GBox equipment (Syngene; Sinoptics, Cambridge, UK) and quantified via densitometry using ImageJ software [33]. The primary antibodies used were the mouse anti-Flag M2 monoclonal antibody (MAb) (Sigma-Aldrich, Burlington, MA, USA) (1:400), or rabbit anti-HA policlonal antibody (Thermo Fisher Scientific, Waltham, MA, USA) (1:1000).

### 2.8. Coimmunoprecipitation Assay

CV1 cell monolayers (corresponding to about 3.5 × 10^5^ cells) were infected with recombinant vaccinia virus vTF7-3, which expresses the T7 RNA polymerase [34]. One hour later, the cells were transfected with 1 µg of pTM1-Z-HA JUNV and 4 µg of pTM1-Flag-NP JUNV. At 24 h post-transfection, the cells were washed with 1× PBS and then lysed in buffer TNE-N (50 mM Tris pH 7.4, 150 mM NaCl, 1 mM EDTA, 0.5 mM dithiothreitol, 0.2% Nonidet P-40) containing protease inhibitors (20 µg/mL phenylmethylsulfonyl fluoride, 50 µg/mL N-tosyl-L-lysine chloromethyl ketone (Sigma-Aldrich, Burlington, MA, USA)). Cell lysates were clarified via centrifugation at 16,000× *g* for 20 min at 4 °C. Aliquots of cytoplasmic extracts were immunoprecipitated with the mouse anti-Flag M2 MAb (Sigma-Aldrich, Burlington, MA, USA) (1:700), or the rabbit anti-HA policlonal antibody (Thermo Fisher Scientific, Waltham, MA, USA) (1:300), using protein A-Sepharose CL-4B Fast Flow (Sigma-Aldrich, Burlington, MA, USA), as previously described [35].

### 2.9. Nuclease Activity Assay

Standard reactions were run in a final volume of 20 µL, with 500 to 1000 nM of wild-type or mutant NP_CTD and the indicated amounts of substrate, in the presence or absence of a cofactor, in a reaction buffer (20 mM Tris-HCl pH 7.5, 150 mM NaCl) at 37 °C for the times indicated. The reaction was stopped by adding EDTA up to a final concentration of 20 mM. The samples were then resolved by electrophoresis on 1% agarose gels containing 0.1 mg/mL Ethidium Bromide.

A single-stranded RNA (ssRNA) substrate was obtained via in vitro transcription. To this, plasmid p5′wt/3′wt_1, which encodes an mRNA analogous to TCRV NP mRNA [36], was linearized through digestion with SmaI, purified via a phenol-chloroform extraction followed by ethanol precipitation, and then used as a template. The reaction was carried out using the MEGAScript^®^ T7 Kit system (Ambion, Austin, TX, USA), and the transcript was purified as previously described [36]. Rotavirus double-stranded RNA (dsRNA), consisting of 11 fragments that ranged from 667 to 3302 bp, was provided by Carlos Palacios (ICT Milstein, Argentina). The molecular weight marker Lambda DNA/HindIII (Pharmacia, Elizabeth, NJ, USA) was used as a double-stranded DNA (dsDNA) substrate.

## 3. Results

### 3.1. Biochemical and Biophysical Characterization of the CTD of JUNV NP

**Figure 1:**
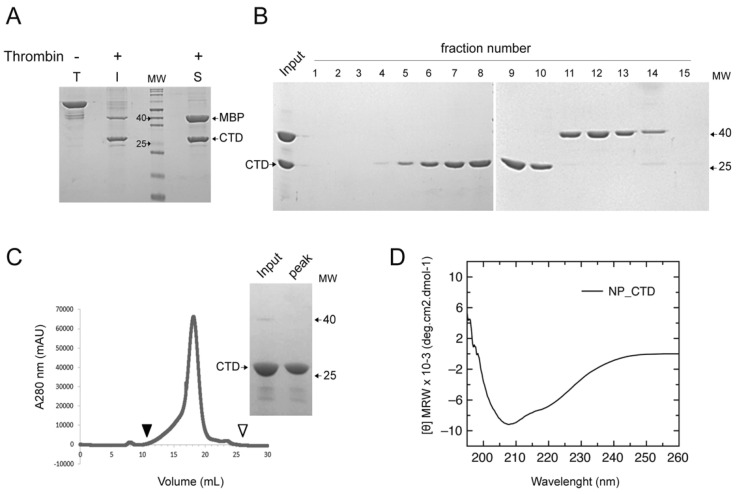
Purification and secondary structure analysis of soluble JUNV NP_CTD. (**A**) SDS-PAGE analysis of MBP-NP_CTD obtained using affinity chromatography; not digested (T), and soluble (S) and insoluble (I) fractions obtained after thrombin digestion. MW, molecular weight markers in kilodaltons (kDa). (**B**) SDS-PAGE analysis of input and eluted fractions (1–15) obtained from a HiTrap Q HP anion exchange chromatography column (Materials and Methods). (**C**) Size exclusion chromatography of purified NP_CTD in a Superdex 200 analytical column; arrowheads indicate the void V0 (filled) and total V0 + Vi (empty) volume of the column. Insert: SDS-PAGE analysis of input and eluted peak fraction. (**D**) Far-UV CD spectrum of purified NP_CTD in 20 mM sodium phosphate pH 7.0.

The sequence of NP_CTD was defined between amino acids 336 and 564 of the NP from the attenuated Candid#1 strain of JUNV, based on the reported crystallographic structure of the full-length LASV NP and amino acid sequence alignment among JUNV, LASV and other arenavirus NPs [16]. The NP_CTD was expressed in bacteria fused to the MBP using the pMAL-NP_CTD recombinant plasmid (Materials and Methods). After purification via affinity chromatography, the fusion MBP–NP_CTD protein was cleaved with bovine thrombin and the soluble NP_CTD was separated from the MBP using anion exchange chromatography (Figure 1A,B).

A biophysical characterization of the JUNV NP_CTD was carried out with the purpose of evaluating the size, conformation, and secondary structure of the protein. The analysis of the highly purified JUNV NP_CTD using size-exclusion chromatography (Materials and Methods) revealed that this domain displays monomeric conformation in its soluble form, with an estimated molecular size of 24 kDa (Figure 1C), consistent with the expected value if globular.

The secondary structure of the NP_CTD was further evaluated using Far-UV CD (Materials and Methods). The Far-UV CD spectrum of a protein results from the combination of the spectral bands corresponding to the different secondary structure elements that compose it. At pH 7.0, the spectrum of the NP_CTD shows minima at ca. 208 nm and 220 nm, consistent with a higher proportion of α-helical content which normally hampers the observation of β-sheet. Indeed, mammarenavirus NP CTD forms a typical α/β/α sandwich architecture, displaying a mixed, five-stranded β-sheet and six α-helices connected by a series of loops [16,21,23]. Overall, these results indicated that the purified NP_CTD displayed the expected size and secondary structure.

### 3.2. JUNV NP_CTD Directly Interacts with JUNV Z

**Figure 2:**
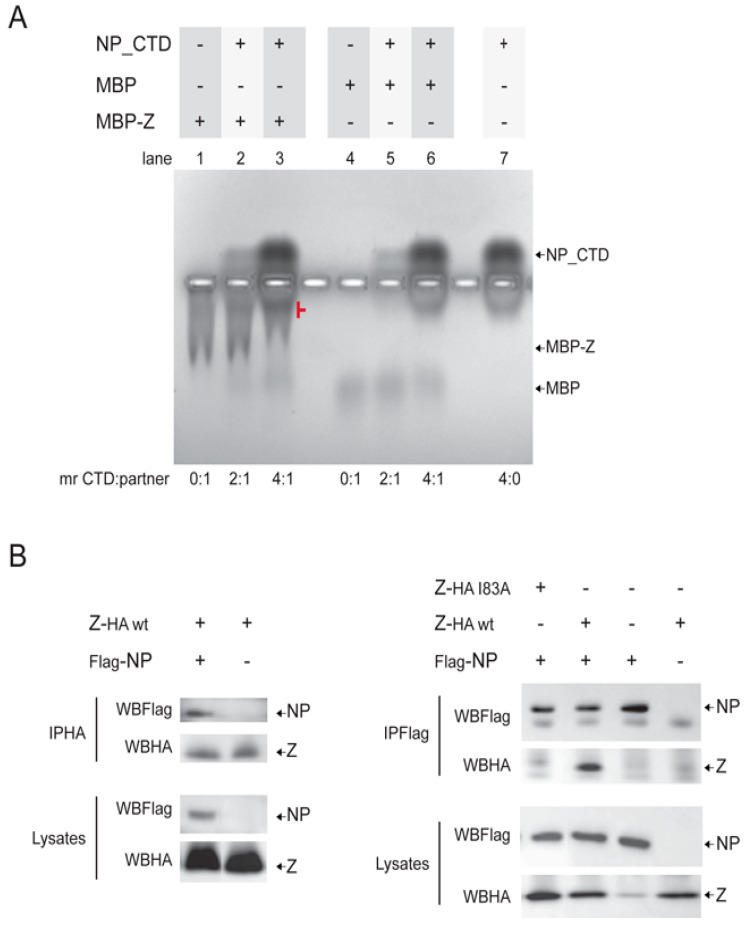
(**A**) Analysis of the interaction between NP_CTD and Z JUNV using electrophoretic mobility shift assay. JUNV NP_CTD was incubated alone or in combination with MBP-Z, or MBP as control, for 40 min at room temperature. In parallel, MBP-Z was incubated alone. Then, soluble and insoluble fractions were separated by centrifugation, and the soluble fraction was analyzed on a native 0.7% agarose gel; proteins were visualized using Coomassie blue staining. For each condition, the molar ratio (mr) of NP_CTD (CTD) to either MBP-Z or MBP (partner) is indicated below the image. (**B**) JUNV NP and Z interact with each other in cellulo. CV1 cells were transfected to express wild-type (wt) or mutant JUNV Z-HA and/or Flag-NP, as indicated. Cell lysates were obtained at 24 h post-transfection and subjected to immunoprecipitation using either anti-Flag or anti-HA serum (IPFlag or IPHA, respectively). Aliquots of the precipitated proteins (IP, upper panels) and aliquots of whole cell lysates (Lysates, lower panels) were analyzed by Western blotting using anti-Flag or anti-HA antibody, as indicated (WBFlag; WBHA).

For several mammarenaviruses, NP and Z proteins were shown to interact with each other, both in infected and transfected cells [9,10,37]. Specifically, in the case of MOPV, TCRV and LCMV, the CTD of NP was reported to play an essential role in the interaction with Z [11,19,38]. Because no information was available about NP–Z interaction for JUNV, we extended the biochemical analysis of the purified protein by examining the ability of the JUNV NP_CTD to bind Z in vitro using an electrophoretic mobility shift assay. To this aim, recombinant JUNV Z was expressed fused to the MBP (MBP-Z, Materials and Methods). JUNV NP_CTD was incubated with the purified MBP-Z, the soluble and insoluble fractions were subsequently separated via centrifugation and the resulting soluble fraction was analyzed on non-denaturing agarose gels (Figure 2A).

The results showed a shift in the MBP-Z JUNV migration after incubation with the NP_CTD, which increased with the increase in the NP_CTD to MBP-Z molar ratio from 2:1 (lane 2) to 4:1 (lane 3; the shifted band is indicated in red). In contrast, no substantial mobility shift was observed at comparable molar ratios for the MBP, included as a control (lanes 4–6). These results suggested that the NP_CTD was able to directly interact with Z JUNV, further supporting that the purified CTD is folded and fully functional. Additionally, we carried out coimmunoprecipitation assays, showing that a Flag-tagged version of the full-length JUNV NP specifically coprecipitated with an HA-tagged version of JUNV Z from cell lysates (Figure 2B). Moreover, a mutant version of JUNV Z carrying change of residue I83 to Alanine displayed a strongly impaired capacity to coprecipitate with the NP, as compared with the wild-type JUNV Z (Figure 2B, right panel). These results supported the specificity of Z–NP binding, indicating that, as previously demonstrated using a TCRV/JUNV chimeric virus-like particle system [9], the C-terminal tail of JUNV Z is important for the interaction with the JUNV NP. Altogether, these findings showed that the JUNV NP interacts with the JUNV Z in mammalian cells, and suggested that the NP CTD mediates this interaction.

### 3.3. JUNV NP_CTD Displays Nuclease Activity

**Figure 3:**
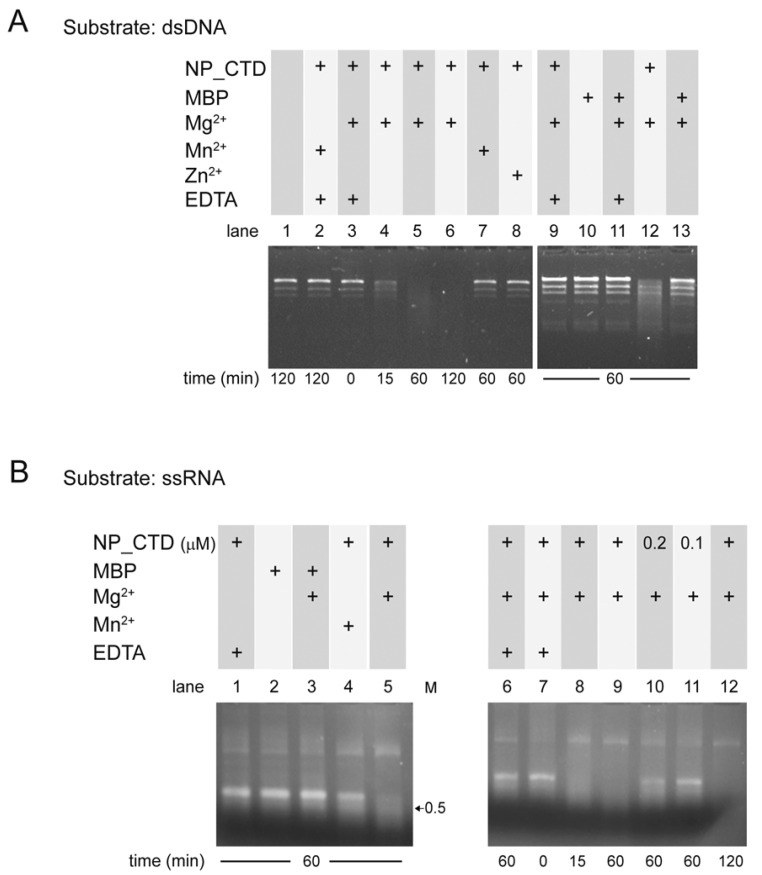
JUNV NP_CTD exhibits nuclease activity. Double-stranded DNA (203 pM, (**A**)) or a 1800 base-long synthetic ssRNA transcript (12 nM, (**B**)) were incubated in the absence or presence of JUNV NP_CTD, or of MBP as control, along with 5 mM of divalent cation with or without 20 mM of EDTA, as indicated. The samples were incubated at 37 °C for the indicated times and the reaction was stopped by the addition of EDTA. The reaction products were resolved in 1% agarose gels containing Ethidium Bromide. Unless otherwise indicated, the concentration of NP_CTD or MBP per reaction was 500 nM. M, the size of a dsRNA marker, is indicated in kilobase pairs.

Previous studies revealed that, despite its structural similarity with the CTD domain of LASV NP, no exonuclease activity could be detected in vitro for the CTD of the JUNV NP [23]. Because our biophysical characterization and mobility shift data (Figure 1 and Figure 2) suggested that the JUNV NP_CTD was purified in a folded conformation, we sought to analyze its nuclease activity in vitro. First, we employed dsDNA as a substrate, using Mg^2+^ or Mn^2+^ as a cofactor. As a control, the substrate was incubated either alone or in the presence of EDTA. The reaction products were analyzed on native agarose gels (Figure 3A).

The results showed that the presence of the NP_CTD and Mg^2+^ was associated with readily detectable dsDNA cleavage after 15 min of incubation (Figure 3A, lane 4, compare with time zero in lane 3) and the increasing degradation of the substrate along the incubation period (lanes 5 and 6). No significative dsDNA degradation was observed after 60 min of incubation when Mn^2+^ or Zn^2+^ were added to the reaction mixture (lanes 7 and 8) or when dsDNA was incubated in the absence of protein (lane 1). The addition of EDTA from time zero to the reaction mixture containing Mg^2+^ (lane 9) prevented substrate degradation. These results indicated that dsDNA cleavage was dependent on the presence of the JUNV NP_CTD and Mg^2+^ in the reaction mixture. This conclusion was further supported by the fact that the MBP, purified from the MBP-NP_CTD (Figure 1), was unable to degrade dsDNA either in the presence or absence of Mg^2+^ (lanes 13 and 10), indicating the absence of bacterial nucleases contaminants during the purification process.

The ability of the JUNV NP_CTD to degrade ssRNA was also evaluated. Incubation of the protein with Mg^2+^ for 60 min, and to a lesser extent with Mn^2+^, led to digestion of the ssRNA substrate (Figure 3B, lanes 5 and 4, respectively). Conversely, no digestion could be observed upon the incubation of the substrate with MBP instead of NP_CTD (lanes 2 and 3). These results suggested that the JUNV NP_CTD can cleave ssRNA and that this activity depends on the presence of divalent cations. Substrate degradation in the presence of Mg^2+^ was readily detected after 15 min of incubation and increased with longer incubation times (compare time 0 in lane 7, with lanes 8, 9 and 12). Partial substrate degradation was detected when the concentration of NP_CTD was reduced by half (lane 10), being almost undetectable when the protein concentration was reduced fivefold (lane 11). No significant ssRNA degradation was observed in the control reactions in the presence of EDTA (lanes 1 and 6).

Overall, these experiments showed that the JUNV NP_CTD displays Mg^2+^-dependent nuclease activity on both dsDNA and ssRNA.

### 3.4. JUNV NP_CTD Nuclease Activity on Double-Stranded RNA Involves the DEDDh Motif

**Figure 4:**
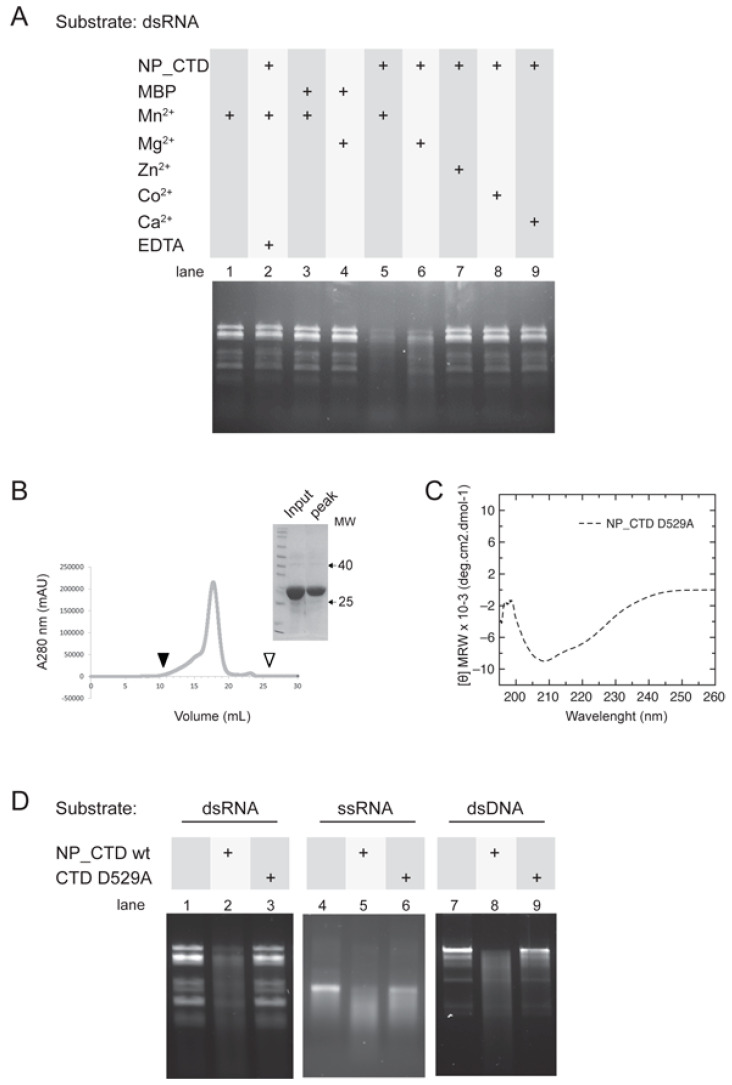
(**A**) NP_CTD degrades dsRNA. Aliquots of human rotavirus dsRNA (339 pM) were incubated in the presence (or absence) of the indicated metal ions, along with 1000 nM of NP_CTD or MBP, at 37 °C for 90 min. As control, the NP_CTD was excluded from the reaction mix (lane 1) or EDTA was added at time zero (lane 2). The reaction was stopped, and the products were resolved using non-denaturing agarose gel electrophoresis, as in Figure 3. (**B**) Analysis of NP_CTD D529A by size-exclusion chromatography on a Superdex 200 analytical column. Arrowheads indicate the void V0 (filled) and total V0 + Vi (empty) volume of the column. Insert: SDS-PAGE analysis of the input and eluted peak fraction. (**C**) Far-UV CD spectrum of the purified NP_CTD D529A in 20 mM sodium phosphate pH 7.0. (**D**) Mutation of the residue D529 impairs NP_CTD nuclease activity. Aliquots of the indicated substrate were incubated in the absence or presence of 1000 nM of either wild-type NP_CTD (NP_CTDwt) or mutant NP_CTD D529A (CTD D529A), along with 5 mM Mn^2+^ (lanes 1–3) or Mg^2+^ (lanes 4–9), for 60 min. The reaction was stopped, and the products were resolved as in Figure 3.

We next evaluated the ability of the JUNV NP_CTD to use dsRNA as a substrate. As shown in Figure 4A, dsRNA was degraded in the presence of Mn^2+^ or Mg^2+^, after incubation with the purified NP_CTD (lanes 5 and 6), but not with the MBP (lanes 3 and 4). Incubation with the NP_CTD and Zn^2+^, Co^2+^ or Ca^2+^ (lanes 7–9) resulted in an undetectable degradation of dsRNA. As expected, no substrate cleavage was observed when dsRNA was incubated with Mn^2+^ in the absence of protein (lane 1) or when it was incubated with both the NP_CTD and EDTA (lane 2). These results indicated that JUNV NP_CTD can digest dsRNA when Mn^2+^ or Mg^2+^ are used as cofactors.

The JUNV NP CTD sequence comprises four strictly conserved acidic residues (D380, E382, D457 and D529) that would conform the putative exonuclease active site [23]. Therefore, to confirm the specificity of JUNV NP_CTD nuclease activity, we expressed and purified a mutant version of the protein carrying change of the residue D529 to Alanine. Characterization of the purified mutant NP_CTD D529A via size-exclusion chromatography indicated that the protein is monomeric in solution, and that it displays an estimated molecular weight (28.2 kDa) close to that expected (Figure 4B). An analysis of the NP_CTD D529A using Far-UV CD showed no change in the spectrum in comparison to the wild-type NP_CTD (Figure 4C), suggesting that the mutation introduced did not affect its folding.

NP_CTD D529A was used for subsequent in vitro nuclease activity assays. Interestingly, the results indicated an undetectable cleavage of dsRNA by the mutant NP_CTD D529A, while, as expected, the wild-type NP_CTD displayed nuclease activity on dsRNA in the presence of divalent cation, after 60 min of incubation (Figure 4D, compare lane 3 to lane 2). In addition, the control showed no substrate degradation in the absence of protein (lane 1). Similarly, the mutant NP_CTD D529A displayed a strongly impaired capacity to cleave either ssRNA (lane 6) or dsDNA (lane 9). These results indicated that the conserved residue D529 of the DEDDh motif is critically important for the nuclease activity of JUNV NP_CTD, and further confirmed that this activity is not the product of a high-activity contaminant.

## 4. Discussion

In this work, the soluble NP_CTD from the attenuated Candid#1 strain of JUNV was purified as a monomer with the expected molecular weight and displaying a secondary structure content that matches the reported structural data of other mammarenavirus NPs [16,21] (Figure 1). The CTD of the JUNV NP was found to bind to Z in vitro, while the full-length JUNV NP and JUNV Z were shown to interact with each other in the cellular context, with residue I83 at the C-tail of Z being involved in this interaction (Figure 2). These results suggest that the NP CTD may be engaged by the Z matrix protein to direct the assembly of JUNV infectious particles, as demonstrated for other mammarenaviruses [9,11,19].

Moreover, our data indicate that the JUNV NP_CTD can cleave dsDNA and has ribonuclease activity on both ssRNA and dsRNA (Figure 3 and Figure 4). In agreement with previous reports on the ExoN activity of the NP from LASV [21,22] and NW Pichinde virus [28], we provide evidence that the JUNV NP_CTD ribonuclease activity on dsRNA is critically dependent on the JUNV homolog residue D529 within the DEDDh motif, and on a divalent metal cofactor (Figure 4). It has been proposed that mammarenavirus NP ExoN activity may contribute to the protein’s ability to block the IFN-I pathway, through the digestion of immune-stimulatory dsRNA as a pathogen-associated molecular pattern molecule (PAMP), generated during virus infection [16,21]. Of note, cell infections with highly pathogenic NW arenaviruses, such as JUNV and Machupo virus (MACV), were recently associated with cytoplasmic dsRNA accumulation. In contrast, no dsRNA was detectable in LASV infection, and the disruption of the LASV NP exonuclease function resulted in dsRNA detection [39,40]. Remarkably, LASV exhibits a strong immunosuppressive effect on the IFN-I pathway [41], while JUNV stimulates the IFN-I response at 24–48 h post-infection at levels that depend on the viral strain [42,43,44]. Whether dsRNAs are degraded by NP early during JUNV infection and, if it was the case, by which mechanism the NP ribonuclease activity is regulated leading to dsRNA accumulation later, remains to be determined. In addition, given the ability of JUNV NP to digest ssRNA, as demonstrated here, it is conceivable that a finely controlled activity of NP ExoN would be required to avoid the cleavage of viral mRNAs. Alternatively, a spatio-temporal separation from NP could operate to protect viral mRNAs from degradation. In this regard, it is worth mentioning that no viral mRNAs could be detected in the NP-containing transcription replication complexes observed in JUNV- and TCRV-infected cells [45].

Besides, the JUNV NP has been shown to disrupt the IFN induction pathway by binding to effector proteins, possibly through the NP CTD [12,13,14,15]. Nevertheless, the relative contribution of these interactions and the ExoN activity to the suppression of the IFN pathway and, particularly, how it is regulated by different strains of JUNV, remains to be elucidated.

So far, two viral families (*Coronaviridae* and *Arenaviridae*) have been reported to encode ExoN proteins that classify into the DEDDh family. SARS-CoV nsp14 hydrolyzes both dsRNA and ssRNA substrates in the 3′-to-5′direction in the presence of Mg^2+^, with a preference for dsRNA substrates [46,47]. Full-length LASV NP degrades dsRNA and dsDNA in the presence of Mn^2+^ and it can also cleave ssRNAs of different sizes in the presence of Mn^2+^ > Co^2+^ > Mg^2+^ [16]. Other studies showed that the LASV NP CTD displays dsRNA specificity in the presence of Mg^2+^ and that it is also active in the presence of Mn^2+^, Co^2+^, and Zn^2+^, although no substantial difference in efficiency between these cations was reported [21]. Furthermore, full-length LASV NP catalytic mutants (D389A, E391A, and D466A) exhibited various degrees of reduction in RNase activity [16]. In line with this, mutant D529A of the JUNV NP_CTD barely maintained some nuclease activity in the presence of Mg2 when either ssRNA or dsDNA was employed as a substrate (Figure 4D). Like nsp14 and other DEDDh proteins, including LASV and MOPV ExoN domains [16,22,25], JUNV NP_CTD ribonuclease activity was undetectable in the presence of Ca^2+^ ions (Figure 4). With dsDNA or ssRNA as substrates, the JUNV NP_CTD appears to preferentially use Mg^2+^ as a cofactor, but it seems to prefer Mn^2+^ to cleave dsRNA (Figure 3 and Figure 4). Other members of the DEDDh family, such as the proofreading domain of many DNA polymerases, most often excise deoxynucleotide substrates in a Mg^2+^ ion-dependent manner [48,49,50], while the Poly(A)-specific ribonuclease (PARN), for example, has been reported to efficiently cleave adenosine trinucleotide in the presence of Mg^2+^, but to hydrolyze adenosine dinucleotide in the presence of Mn^2+^ [51]. Thus, it is possible that the nature of the substrate might modulate the choice of the divalent metal ion used as a cofactor for the JUNV NP_CTD, influencing the stability of the enzyme–substrate complex and the catalytic efficiency. Further investigations are needed to thoroughly define the contribution of individual residues to the nuclease activity, as well as substrate and cation preferences for the JUNV NP_CTD.

The nsp14 of coronaviruses is known to possess proof-reading activity and to play an important role in anti-IFN function [52,53]. It would be interesting to evaluate whether the ExoN activity of the NP from mammarenaviruses may be involved in an RNA proof-reading function as well, as recently suggested for the NP from MOPV and LCMV [54]. In sum, this work contributes to the understanding of the JUNV NP functions and highlights the relevance of the DEDDh motif of this protein, which, as previously shown for SARS-CoV-2 and OW LASV and LCMV [55,56,57], might be evaluated as potential target for the design of antiviral drugs to combat AHF, a concept that could be extended to other pathogenic NW mammarenaviruses.

## Figures and Tables

**Table 1:** Names and sequences (from 5’ to 3’) of the oligonucleotides used in PCR. The ATG codon is indicated in bold italics. The point change introduced into the viral sequence via site-directed mutagenesis is red-colored.

Oligonucleotide Name	Sequence 5′ to 3′
CTD-NJunv Fw	C***ATG***AAACCAGTTGCTGGTCCTAGACAG
NJunv Rv	TAGAGAATTCTTATCACAGTGCATAGGCTGCCTTCGG
Flag-NJunv Fw	C***ATG***GATTACAAGGATGACGACGATAAGGCACACTCCAAAGAGGTTCCAAGC
NJunvD529A Fw	CACTGTGCTCTGCTAG**C**CTGCATAATGTTTCAGTC
NJunvD529A Rv	GACTGAAACATTATGCAG**G**CTAGCAGAGCACAGTG

## Data Availability

Data are contained within the article.
